# Recurrent left main stem stenosis in a young female with Behçet’s aortitis: a case report

**DOI:** 10.1093/ehjcr/ytae331

**Published:** 2024-07-11

**Authors:** Sotirios Dardas, Ibrahim Antoun, Falik Sher, Navid Munir, Kosmas Kontoprias

**Affiliations:** Department of Cardiology, University Hospitals of Derby and Burton NHS Foundation Trust, Royal Derby Hospital, Uttoxeter Rd, Derby, DE22 3NE, UK; Department of Cardiology, University Hospitals of Derby and Burton NHS Foundation Trust, Royal Derby Hospital, Uttoxeter Rd, Derby, DE22 3NE, UK; Department of Cardiovascular Sciences, University of Leicester, Leicester, UK; Department of Cardiology, University Hospitals of Derby and Burton NHS Foundation Trust, Royal Derby Hospital, Uttoxeter Rd, Derby, DE22 3NE, UK; Department of Cardiology, University Hospitals of Derby and Burton NHS Foundation Trust, Royal Derby Hospital, Uttoxeter Rd, Derby, DE22 3NE, UK; Department of Cardiology, University Hospitals of Derby and Burton NHS Foundation Trust, Royal Derby Hospital, Uttoxeter Rd, Derby, DE22 3NE, UK

## Abstract

**Background:**

Behçet’s disease (BD) is a rare and complex vasculitis disorder renowned for its diverse clinical presentations. Cardiovascular involvement is reported to be present in 7–46% of the patients, with coronary arteries being involved in only 0.5%. The management of cardiovascular complications can be challenging due to the rarity of such cases and the absence of standardized guidelines regarding diagnosis and treatment.

**Case summary:**

We report the case of a 27-year-old patient with BD with known aortitis and pulmonary arteritis, who presented with recurrent acute coronary syndromes related to critical left main coronary artery stenosis. She was initially managed with percutaneous coronary interventions twice. Following recurrent stent failure, she eventually underwent urgent coronary artery bypass surgery, together with aortic valve replacement and aortic root repair. She made an uneventful recovery and remains well 6 months following her operation.

**Discussion:**

This case illustrates the significant challenges that can be encountered when managing coronary complications in patients with BD. Both percutaneous and surgical options have been reported in the literature with variable outcomes. Multi-disciplinary team involvement is of utmost importance in order to offer a balanced therapeutic strategy to these patients. Further research is required to shed light to the unknowns surrounding this rare cohort.

**Learning points**

- Coronary artery involvement in Behçet’s disease (BD) is rare. When present, it is associated with significant and potentially life-threatening complications.
- Treatment of coronary complications in patients with BD can have unpredictable outcomes, and the lack of standardized guidelines makes their management challenging.
- Multi-disciplinary team approach is of utmost importance when managing patients with BD and cardiovascular involvement.

## Introduction

Atherosclerotic coronary artery disease (CAD) is by far the commonest cause of acute coronary syndromes (ACS). Traditional risk factors play a major role in the development of atherosclerosis. However, there are other non-atherosclerotic causes of CAD, including inflammation.

Behçet’s disease (BD) is a rare and complex vasculitis disorder renowned for its diverse clinical presentations, including mucocutaneous lesions, uveitis and arthritis. The relationship between BD and cardiovascular complications underscores the importance of early recognition and vigilant monitoring. However, cardiovascular involvement can be challenging to detect, given its diverse clinical presentation that can mimic other conditions. Coronary involvement is uncommon (reported prevalence of 0.5%) and can pose significant diagnostic and therapeutic challenges.^1^

We describe the case of a young female with BD who presented with recurrent ACS. She was treated with percutaneous coronary intervention (PCI) twice and eventually with coronary artery bypass graft (CABG), together with aortic valve (AV) replacement and aortic root repair, making a good recovery.

## Summary figure

**Table:** 

Timeline of critical events	
Month 0	A 27-year-old female presented to our hospital with pyrexia, orogenital ulcers and chest pain. A computed tomography (CT) scan of the aorta showed dilated aortic root, and cardiac magnetic resonance (CMR) imaging demonstrated active aortitis and pulmonary arteritis. Mild aortic regurgitation (AR) was also noted. The diagnosis of Behçet’s disease was made, and she was started on immunosuppressive therapy.
Month 4	The patient represented with an ACS. Left heart catheterization (LHC) revealed severe ostial left main stem (LMS) artery disease. Following Heart Team discussion, intravascular ultrasound (IVUS)-guided PCI was employed with excellent result.
Month 13	The patient presented for the third time with symptoms suggestive of unstable angina. Left heart catheterization revealed severe in-stent restenosis (ISR) of the LMS involving the ostium of the left anterior descending artery (LAD). She was managed with repeat IVUS-guided PCI with deployment of a drug-eluting stent (DES) in the LMS/LAD arteries overlapping with the previous stent. The result was excellent again.
Month 22	The patient represented for the fourth time with crescendo angina. Left heart catheterization showed severe ISR, this time involving the coronary ostia of both the LAD and circumflex arteries. Given the recurrent stent failure, she was referred for urgent CABG.
Month 22	The patient underwent CABG together with AV replacement and aortic root repair, due to progression of the AR. She had an uneventful recovery and was discharged on the sixth postoperative day.
Month 28	The patient remains well and asymptomatic 6 months following her surgery. She continues with regular infliximab infusions and corticosteroids under the care of the rheumatology team.

## Case presentation

A 27-year-old white female presented to our hospital with a week’s history of crescendo angina and intermittent chest pain at rest. Physical examination findings were normal. This was her *fourth admission* within 2 years.

**Figure 1:**
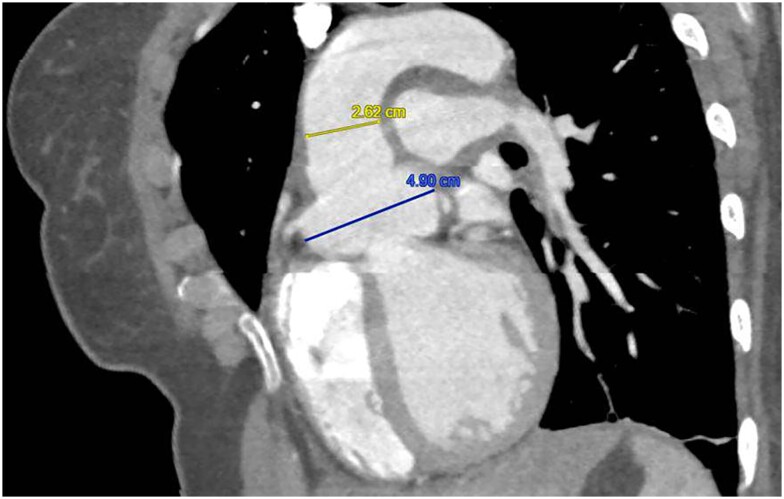
Computed tomography aorta in sagittal view demonstrating aneurysmal dilatation of the right and left sinuses of Valsalva with aortic root diameter of 4.9 cm. The proximal ascending aorta measured normal at 2.62 cm; 94 × 149 mm.

**Figure 2:**
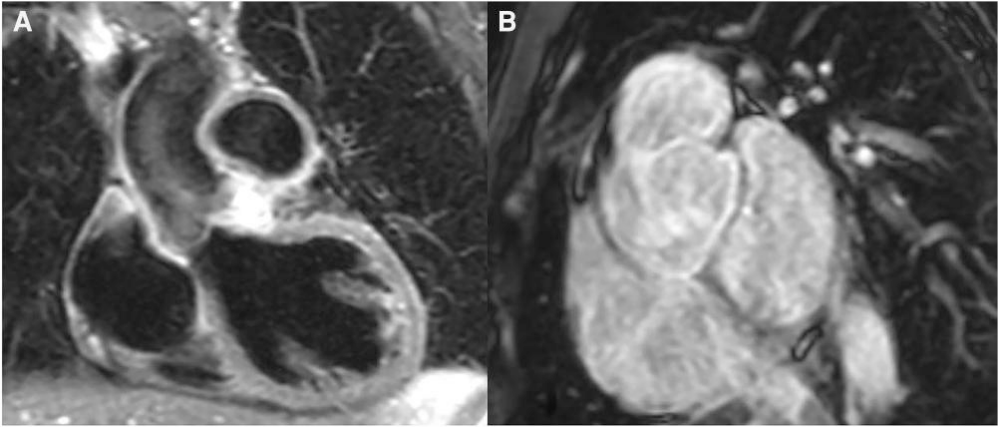
(*A*) Cardiac magnetic resonance demonstrating circumferential wall thickening and increased signal intensity on short tau inversion recovery imaging indicative of active aortitis and pulmonary arteritis. (*B*) Cardiac magnetic resonance demonstrating circumferential late gadolinium enhancement of the aortic root and main pulmonary artery; 91 × 214 mm.

She had a background of BD diagnosed 2 years ago, following her *first admission* with pyrexia, orogenital ulcers and chest pain. At the time, her laboratory tests showed raised inflammatory markers, including erythrocyte sedimentation rate and C-reactive protein, as well as neutrophil leucocytosis. A CT of the aorta revealed aneurysmal dilatation of the right and left sinuses of Valsalva (SoV) with aortic root diameter of 4.9 cm (*Figure 1*). The right subclavian and axillary arteries showed mild aneurysmal dilatation as well. Cardiac magnetic resonance demonstrated active aortitis, evidenced by circumferential wall thickening and increased signal intensity around the dilated SoV and the ascending aorta on T_2_ short tau inversion recovery (STIR) imaging. Inflammation was also present in the main pulmonary artery and its branches. Circumferential late gadolinium enhancement was also noted in the above territories (*Figure 2*). Mild AR was present as well. Her initial International Criteria for BD (ICBD) 2014 score was 5, given the oral and genital ulceration and vascular involvement. She was treated initially with cyclophosphamide and corticosteroids, followed by infliximab infusions every 6 weeks.

**Figure 3:**
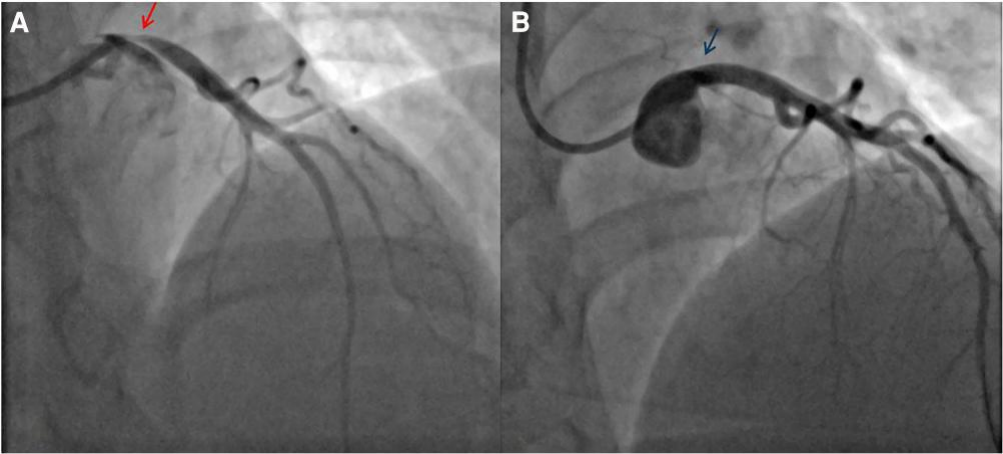
(*A*) First coronary angiogram demonstrating critical ostial left main stem stenosis (arrow). (*B*) Angiographic result after first percutaneous coronary intervention (arrow); 102 × 226 mm.

Four months after the BD diagnosis, the patient had her *second admission*. This was due to an ACS, for which LHC revealed severe localized ostial LMS stenosis due to a fibrotic lesion, as demonstrated in the IVUS. Given the recent diagnosis of BD and concerns of ongoing inflammation, following surgical consultation, PCI was deemed the safest option. Therefore, she underwent PCI with a 4.5 × 12 mm Synergy Megatron DES with excellent results (*Figure 3*). Her ICBD 2014 score during this admission had reduced with the infliximab treatment to 1, accounting for the vascular involvement. She was discharged on dual antiplatelet therapy with aspirin and ticagrelor, together with standard secondary prevention medications including a statin and a beta-blocker.

**Figure 4:**
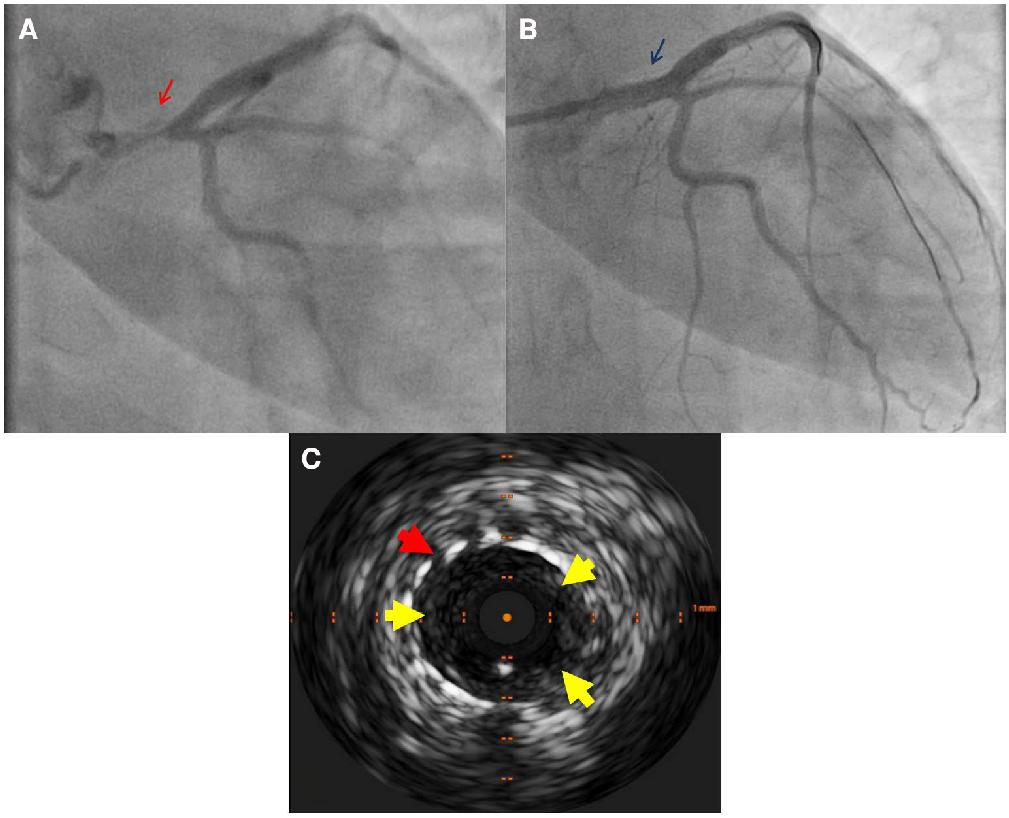
(*A*) Second coronary angiogram demonstrating severe in-stent restenosis of the first left main stem drug-eluting stent, involving the left anterior descending artery ostium (arrow). (*B*) Angiographic result after the second percutaneous coronary intervention (arrow). (*C*) Intravascular ultrasound demonstrating well-apposed stent in the left main stem (outer arrow) with significant neointimal formation (inner arrows); 187 × 232 mm.

The patient had her *third admission* after 9 months with unstable angina, for which LHC demonstrated severe ISR of the LMS stent, extending into the ostium of the LAD. Intravascular ultrasound showed significant neointimal formation. This was managed with PCI to the LMS/LAD with a 4.0 × 16 mm Synergy DES overlapping with the pre-existing LMS stent, achieving an excellent result (*Figure 4*). Her ICBD 2014 score had remained 1 accounting for the vascular involvement, and the advice from the rheumatology team was to continue with infliximab infusion. She also continued on standard post-ACS therapy as previously.

**Figure 5:**
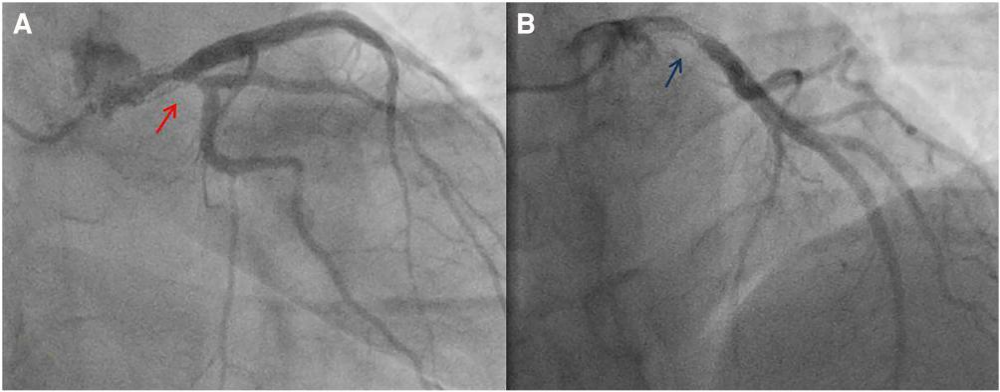
(*A*) Third coronary angiogram in caudal view demonstrating recurrent severe in-stent restenosis, involving the ostia of the left anterior descending and left circumflex arteries (arrow). (*B*) Third coronary angiogram in cranial view demonstrating the recurrent severe in-stent restenosis (arrow); 79 × 203 mm.

Returning to her *fourth admission*, given her background and mildly raised troponin (19 ng/L), the patient was taken for repeat LHC, which again revealed severe ISR, this time also involving the left circumflex artery ostium (*Figure 5*). A repeat IVUS was not performed on this occasion, as it was felt that the mechanism of stent failure was already established and there was no intention to proceed to PCI this time. Because of the recurrent stent failure and following discussion with the patient, she was referred for urgent CABG. Pre-operative transoesophageal echocardiogram demonstrated AR progression to moderate to severe. Intraoperative findings revealed thickened AV leaflets with mild calcification. The aortic wall was also thickened in keeping with vasculitis. The AV thickening together with the aortic root dilatation was felt to be the main reason for significant AR. The patient underwent CABG with left internal mammary artery anastomosed to the mid-LAD and a saphenous vein graft anastomosed to the first obtuse marginal branch. Aortic valve was replaced with a 25 mm Resilia bioprosthesis, and the aortic root was repaired using a 36 mm tube graft. It is worth mentioning that her ICBD 2014 score had remained 1 (accounting for the vascular involvement) on this occasion as well.

She had an uneventful recovery and was discharged home on the sixth postoperative day with a plan to complete 12 months of dual antiplatelet therapy, followed by long-term aspirin and standard secondary prevention medications. She remains well and asymptomatic 6 months following her operation. She continues with regular infliximab infusions and corticosteroids under the care of the rheumatology team.

## Discussion

Cardiovascular involvement in BD varies from 7 to 46%.^2^ It can manifest as arterial stenosis, thrombosis or aneurysmal formation, leading to potentially life-threatening complications, including ACS, myocarditis, cardiomyopathy, valvular dysfunction, intracardiac thrombi and aneurysmal formation of the aorta and its branches.^3^ The mortality rate was 13.5% in a series of 101 patients, suggesting a poor prognosis.^4^ The aorta is among the most affected arteries, followed by the pulmonary artery. It mainly manifests as SoV aneurysm and aortitis.^5,6^

The proposed mechanism is vasculitis with a T lymphocyte-mediated immune reaction causing neutrophilic attraction, eventually leading to vessel wall destruction and aneurysm formation.^5^ Another hypothesis includes endothelial dysfunction activating a cascade of events that lead to abnormal thrombo-coagulation and vasculitis.^7^

Coronary involvement can present as vasculitis-induced aneurysms, thrombosis or stenosis, leading to life-threatening events, including ACS.^8^ Coronary occlusion is attributed to fibrous intimal thickening related to local vasculitis.^9^

Several small-scale studies have reported coronary artery involvement in patients with BD, emphasizing the importance of heightened clinical suspicion and meticulous evaluation in individuals presenting with chest pain, atypical symptoms of myocardial ischaemia or signs of ACS in this cohort.^6,8^ This was supported in a systematic review including 62 BD patients, which revealed that ACS in BD tends to affect young males with a low prevalence of cardiovascular risk factors.^10^

The diagnosis of coronary artery involvement in BD demands a multi-disciplinary approach, often integrating clinical findings, imaging modalities and laboratory investigations. Imaging techniques, such as coronary and CT angiography, are invaluable in detecting and characterizing coronary lesions associated with vasculitis in BD.^11^

Managing coronary artery complications in BD poses significant therapeutic challenges due to the rarity of such cases and the absence of standardized treatment guidelines. Treatment strategies typically involve a combination of immunosuppressive agents, including corticosteroids, azathioprine, cyclophosphamide and occasionally biological agents like anti-TNF drugs, to mitigate inflammation and prevent further vascular damage.^12^ Patients presenting with ACS have previously been treated with PCI and CABG. Experience and long-term outcomes are limited, and both approaches have their limitations. Percutaneous coronary intervention carries an increased risk of ISR and thrombosis.^13,14^ Surgery can also be problematic with the development of pseudo-aneurysms and, therefore, is not recommended in the acute phase of the disease.^15^

Despite the challenges, elucidating the intricacies of coronary artery involvement in BD remains a point of ongoing research. Advances in understanding the underlying pathophysiology and identifying more targeted therapeutic approaches hold promise for improving outcomes and enhancing the management of this unique and intricate facet of BD.

To our best knowledge, our case is unique in that it reports a combination of both great vessel and coronary involvement in a young person with BD. In our case, the patient presented with recurrent LMS stenosis in the absence of coronary aneurysms, which are usually described alongside coronary stenoses in patients with BD.

In conclusion, while coronary artery involvement in BD is uncommon, its potential for severe and life-threatening complications necessitates high clinical awareness, early recognition and tailored therapeutic interventions, as it affects younger individuals. Collaborative efforts between clinicians and researchers are imperative in unravelling the complexities surrounding this manifestation of BD, ultimately paving the way for more effective management strategies and improved patient outcomes.

## Lead author biography


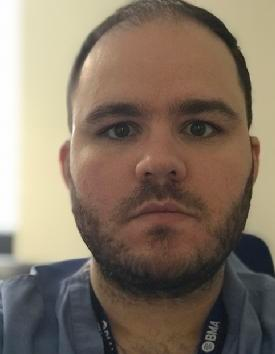


Dr Sotirios Dardas graduated from the Medical School of the Aristotle University of Thessaloniki, Greece, in 2014. He subsequently moved in the UK for his postgraduate training. He is currently a Cardiology Specialty Trainee in the East Midlands Deanery. His career aspiration is to become a specialist in coronary and structural heart interventions. He is due to start a 2-year Interventional Cardiology Fellowship in London, ON, Canada, in July 2024.

**Consent:** Consent for publication has been obtained from the patient, in line with the COPE best practice.

**Funding:** None declared.

## Data Availability

No new data were generated or analysed in support of this research.
